# Insights into perceptions, responses, and challenges experienced by women and girls’ survivors of sexual violence and their communities in rural Guinea, 2020

**DOI:** 10.3389/fgwh.2024.1365601

**Published:** 2024-11-01

**Authors:** Delphin Kolié, Abdoulaye Sow, Graziella Ghesquiere, Stefaan Van Bastelaere, Maurice Sandouno, Thierno Souleymane Diallo, Sabine Soropogui, Yaya Barry, Thierno Oumar Fofana, Bienvenu Salim Camara, Sidikiba Sidibé, Thérèse Delvaux, Alexandre Delamou

**Affiliations:** ^1^ Ministry of Health and Public Hygiene, National Research and Training Centre in Rural Health of Maferinyah, Forécariah, Guinea; ^2^ Centre of Excellence for the Prevention and Control of Communicable Diseases, University of Conakry, Conakry, Guinea; ^3^ Fraternité Médicale Guinée, Conakry, Guinée; ^4^ Belgium Development Agency (ENABEL), Guinea Office, Conakry, Guinea; ^5^ Belgium Development Agency (ENABEL), Brussels, Belgium; ^6^ Ministry of Health and Public Hygiene, Health District Directorate of Mamou, Mamou, Guinea; ^7^ Ministry of Health and Public Hygiene, Health District Directorate of Télimélé, Télimélé, Guinea; ^8^ Department of Public Health, Institute of Tropical Medicine of Antwerp, Antwerp, Belgium

**Keywords:** perceptions, responses, challenges, sexual violence, women and girls, Guinea, mix-methods research

## Abstract

**Introduction:**

Gender-based violence (GBV), particularly sexual violence, is a significant global public health issue with severe physical, psychological, and social consequences for survivors and their communities, especially among women and girls. In Guinea, limited data exist on the frequency and management of sexual violence in rural areas. This study aimed to analyze the perceptions, responses, and challenges faced by women and girls’ survivors of sexual violence and their communities in two rural districts of Guinea in 2020.

**Methods:**

A parallel mixed-methods approach was employed, integrating quantitative and qualitative data. For the quantitative analysis, all reported cases of GBV from public health facilities and directorates of girls and women’ promotion were collected from January 1 to December 31, 2020 in the health districts of Télimélé and Mamou. The qualitative component involved key informant interviews with four main participant groups: survivors of GBV and their support networks, healthcare providers, stakeholders and partners involved in GBV prevention and response, and community leaders. Data were analyzed to identify patterns in case reporting, perceptions of violence, responses by survivors and communities, and challenges to effective management.

**Results:**

The study revealed a high frequency of reported sexual violence among women, with substantial disparities between the two districts. In Mamou, sexual violence among women constituted 61% of all reported GBV cases, whereas in Télimélé, it accounted for only 8%. Additionally, data on sexual violence were inconsistent, with discrepancies in terminology and significant underreporting of cases. Survivors and their families predominantly sought conciliation with perpetrators’ families, motivated by fear of retaliation, social stigmatization, and exclusion. This response was more prevalent in communities with limited law enforcement, where perpetrators were often released after short periods of detention. Participants highlighted several barriers to accessing health services and providing comprehensive care to survivors. These barriers included socio-economic constraints, a lack of skilled healthcare providers, frequent stock-outs of essential medical supplies, and the absence of psycho-social and legal support at the community level.

**Conclusion:**

The findings highlight the urgent need to enhance the capacity of decentralized health and social services to deliver integrated, patient-centered care for sexual violence. There is also a pressing need for stronger enforcement of laws related to sexual violence, enhanced training for healthcare providers, and the harmonization of GBV data reporting tools. Tackling socio-cultural barriers through community education, while enhancing access to legal and psychological support are crucial for reducing the frequency of sexual violence and ensuring timely, quality care for survivors in Guinea.

## Introduction

Sexual violence is known as any sexual attempt or act against one's sexuality using harassment, intimidation, or physical force, irrespective of the relationship with the victim (intimate partner or not) (1). Sexual violence is a global public health concern because of its frequency, increasing magnitude, and consequences on survivors and their surroundings (2, 3). For example, the prevalence of physical and/or sexual violence by an intimate partner increased worldwide from 30% to 37% between 2013 and 2018 (4, 5). Whilst sexual violence affects both sexes, women and girls are most affected by the phenomena (6). They also are at greater risk of depression, anxiety, post-traumatic stress and suicidal behaviour following sexual violence (7–10). Moreover, female survivors of sexual violence are exposed to sexually transmitted infections including HIV/AIDS, unwanted pregnancies, and unsafe abortions; these negatively affect their health and development prospects (6, 8, 11).

The frequency of sexual violence is underestimated due to a lack of appropriate care and survivors’ fear of stigmatisation (6, 12). According to recent estimates, approximately 27% of women worldwide are sexually abused during childhood (5). Also, 38% of worldwide women murders are committed by an intimate partner or acquaintances, compared with 6% for men (5). Nevertheless, the burden sexual violence varies across the world. The prevalence of sexual violence in both sexes is estimated at 33% in sub-Saharan Africa (SSA) compared with 22% in Europe and 25% in the Americas (5). Sexual violence is widespread in SSA (13–17). In Ethiopia, workplace sexual violence is estimated at 22%, including attempted rape (14%) rape (8%), and harassment (33%) (18). In South Africa, 21% of sexual violence is reported among teenage girls and aggressors use coercion, force or threats to perpetrate the violence (16).

Factors contributing to the risk of sexual violence against young girls and women are wide-ranging and include early loss of a parent, previous experience of aggression, poverty, hazardous alcohol or drug use, physical disabilities, and exposure to domestic violence (19–23). While these factors are informative, they do not directly inform prevention interventions, particularly since many survivors choose not to disclose their assaults (17, 21, 24, 25). Consequently, understanding community perceptions and responses to sexual violence is vital for delivering effective prevention strategies tailored to young girls and women.

However, in many communities, the dominant social response to sexual violence is characterized by stigma, silence, and cultural norms that prioritize preserving family honor over justice for the survivor (26–29). This environment fosters hostility toward survivors, where fear of ostracism or retaliation often discourages them from disclosing their experiences (30–32). Consequently, many communities internalize these societal pressures, leading to underreporting and a hesitancy to access critical support services such as healthcare, psychological counseling, or legal assistance (30–32). Research into community perceptions and responses to sexual violence is essential due to the complex interplay between societal responses to sexual abuse and survivors’ willingness or ability to seek help (32).

In Guinea, the prevalence of gender-based violence (GBV) among girls and women was estimated at 46% in 2013 (33). However, there is limited knowledge regarding the proportion of sexual violence within the broader category of GBV. Additionally, the responses adopted by survivors of sexual violence, their families, and the community remain largely unexplored. To the best of our knowledge, no study has investigated community perceptions, challenges, and responses to sexual violence in Guinea. A nuanced understanding of these dynamics is essential to developing effective, culturally appropriate strategies that encourage survivors to disclose their experiences, facilitate access to healthcare, and disrupt the cycle of silence and impunity surrounding sexual violence. For instance, in rural communities of the Democratic Republic of Congo, a common response to sexual violence was the forced marriage of survivors to their aggressors (34, 35). This practice catalyzed the development of a national strategy against sexual and GBV in 2009, aimed at strengthening law enforcement and enhancing the quality of healthcare provided to survivors (34, 35). Furthermore, such research holds the potential to inform broader policy initiatives aimed at enhancing legal frameworks, community-based interventions, and health systems to better meet the needs of survivors of sexual violence in Guinea.

Hence, this study had four main objectives:

• Determine the proportion of sexual violence among women and girls within the broader spectrum of GBV.
• Analyze the perceptions of women and girls’ survivors and their communities regarding the recurrence, severity, and contributing factors of sexual violence.
• Explore the responses implemented at the individual, family, community, and health system levels in addressing sexual violence among women and girls.
• Examine the challenges encountered by survivors and their surroundings at individual, community, and health system levels in combating sexual violence among women and girls.

## Methods

### Study design

This was a parallel mixed-methods study using qualitative and quantitative approaches (36). The quantitative component estimated the proportion of sexual violence within the broader category of GBV using two distinct sources: the district health information system 2 and the registries of the Ministry of gender and women’ promotion.

The qualitative component of the study explored the perceptions, responses, and challenges faced by women and girls’ survivors of sexual violence and their communities.

### Study setting

Guinea, located in West Africa, had an estimated population of 12 million in 2020. The country shares borders with six nations, including Sierra Leone, Senegal and Mali, all of which display high rates of GBV (5). According to 2017 census data, the regions of Kindia and Mamou are among those most affected by GBV in Guinea (37). However, these data were not stratified to reflect the specific districts within these regions (37). Additionally, the proportion of sexual violence within the broader category of GBV remained undocumented (37).

Public response mechanisms for addressing sexual violence in Guinea involve five entities: the security department, represented by the office for the protection of gender, children and morals; the judicial system (courts); civil society organizations; healthcare facilities; and the department of gender and women’ promotion.

The study was conducted in two districts: Télimélé and Mamou. In 2020, Télimélé had a population of 339,969 while Mamou had 382,221 inhabitants (38). The local healthcare system in Télimélé includes one district hospital, 15 health centres (including one urban center), and two officially recognized private clinics. In contrast, Mamou features one regional hospital, 18 health centres (including five urban centers) and four officially recognized private clinics.

### Study sources and participants

The quantitative data were sourced from the district health information system (an electronic database) and were contrasted with data from the directorates of gender and women’ promotion to understand the patterns and trends of GBV in the region. This comparison aimed to provide a comprehensive overview of the frequency of GBV, facilitating the identification of gaps in service provision and areas for improvement in public health interventions. By integrating these data sources, we sought to gain insights into the relationship between health service accessibility and community responses to GBV, ultimately informing more effective strategies for addressing the needs of survivors. These data covered the period from January 1 and December 31, 2020. These data were compiled using an Excel spreadsheet. It is important to note that the records maintained by the directorates of gender and women’ promotion also include information from police and court systems; thus, targeting this directorate for data collection was essential.

For the qualitative component, four primary participant groups were targeted: (1) survivors of sexual violence and/or their relatives; (2) healthcare providers working in various public (hospitals, health centers) and private health facilities, including community relays; (3) partners and stakeholders engaged in combating GBV at the district level, such as representatives from the directorate of gender and women’ promotion, NGOs, and security and justice officials; and (4) community leaders, including religious leaders and neighborhood chiefs. Qualitative data were gathered through in-depth individual interviews, guided by a structured interview protocol and conducted by a pool of experienced researchers familiar with the local context and qualitative methods. These interviews were recorded using Android phones to ensure the accurate capture of participants’ responses, facilitating thorough analysis and interpretation of the data collected.

Participants to qualitative component were selected using a stratified sampling technique to ensure diversity and representation across the identified groups. The first step involved delineating the four participant categories. Subsequent selection aimed to achieve maximum variation within and among these groups, allowing for a comprehensive exploration of perceptions and experiences related to sexual violence. Survivors were identified by community health workers and were interviewed in convenient, private locations to maintain confidentiality and encourage open communication regarding their experiences. This approach not only ensured the richness of the data collected but also adhered to ethical principles of respect and care for participants’ well-being.

### Data analysis

A comparison of the number of reported cases of sexual violence and the proportion of sexual violence relative to all reported GBV cases was conducted between the study sites and various data sources, utilizing an Excel database.

For the qualitative data, an analysis grid was developed in alignment with the study objectives. Interviews were transcribed and manually coded, employing a thematic analysis that integrated both inductive and deductive approaches (39). All interviews were independently coded by DK and BSC over a two-week period, with any discrepancies resolved through team consultations.

The diversification of study participants and methods, encompassing both quantitative and qualitative data, facilitated the triangulation of findings, thereby enhancing the internal validity of the study. This comprehensive methodological approach allowed for a more nuanced understanding of the complexities surrounding sexual violence in the study context.

### Ethical considerations

The study protocol was approved by the National Ethics Committee for Health Research of Guinea, under the reference number 061/CNERS/21. Participation in this study was voluntary and conducted with informed verbal consent obtained from all participants prior to the interviews. The collected information was stored on a computer with access restricted solely to research investigators. Interview files were pseudonymized, and each participant was assigned a unique ID code to ensure confidentiality and protect their identity throughout the research process.

## Results

### Reported cases of sexual violence (quantitative component)

**Figure 1:**
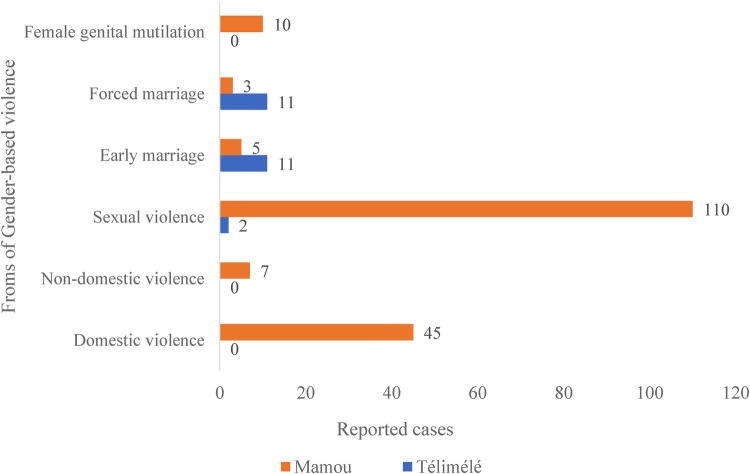
Distribution of gender-based violence by type in the districts of Télimélé and Mamou, 2020 (*N = 204*); data sourced from the directorates of gender and women’ promotion.

**Figure 2:**
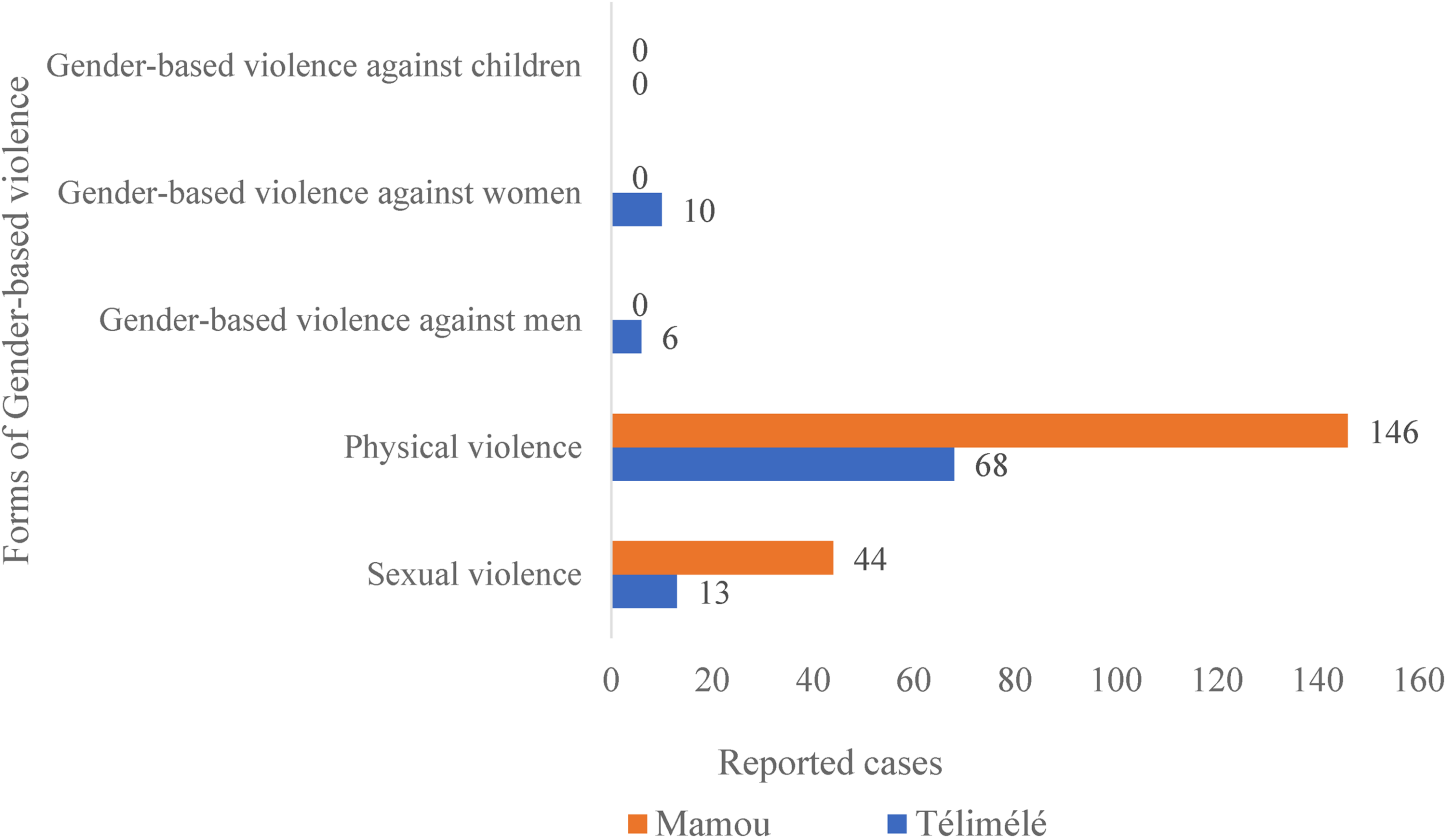
Distribution of gender-based violence by type in the districts of Télimélé and Mamou, 2020 (*N = 287*); data sourced from district health information system 2 (DHIS2).

Figures 1, 2 below summarize the frequencies and typology of sexual violence recorded in the Télimélé and Mamou districts in Guinea for the year 2020.

A total of 112 cases of sexual violence were reported by the directorates of gender and women’ promotion in 2020, representing 55% of all reported GBV cases (*n = 204*) in the two selected districts. The distribution of reported sexual violence cases varied significantly between the study districts. Specifically, in the Mamou district, sexual violence accounted for 61% (110 out of 180) of all GBV cases, while in the Télimélé district, it constituted only 8% (2 out of 24) (see Figure 1).

Data from the district health information system (DHIS2) in 2020 indicated that sexual violence represented 20% (57 out of 287) of all recorded GBV cases in the Télimélé and Mamou districts. The proportions varied further by district, with 14% for Télimélé and 23% for Mamou.

An analysis of sexual violence cases by reporting sources, specifically the directorates for gender and women’ promotion and DHIS2, revealed discrepancies in both terminology and reporting frequencies across the districts. For instance, DHIS2 categorizes incidents using terms such as sexual violence, physical violence, and GBV against women, men, and children. In contrast, the directorates of gender and women’ promotion use classifications that include domestic and non-domestic violence, early and forced marriage, sexual violence, and female genital mutilation. This indicates that while both sources address sexual violence, they utilize different nomenclature and encompass varying categories of victims, which can impact data consistency and interpretation.

### Perceptions, responses, and challenges experienced by stakeholders regarding sexual violence (qualitative component)

A total of 34 in-depth individual interviews were conducted across the two study districts: 14 in Télimélé and 20 in Mamou. Table 1 below presents the characteristics of the participants.

**Table 1:** Characteristics of participants.

Characteristics	Télimélé		Mamou		Total (%)
	Female	Male	Female	Male	
Groups of participants					
Survivors of sexual violence	5	0	4	0	9 (25)
Health care providers	0	1	2	4	7 (21)
Community leaders					
Religious leader	0	1	0	1	2 (6)
Neighbourhood chief	0	1	0	1	2 (6)
Other stakeholders					
Security	0	2	0	2	4 (12)
Gender and women’ promotion	1	0	0	2	3 (9)
NGO	2	1	2	1	6 (18)
Justice (Court)	0	0	0	1	1 (3)
Total	8	6	8	12	34

#### Stakeholders’ perceptions of sexual violence

Participants’ expressed concerns regarding the increasing prevalence of sexual violence in their communities, emphasizing its severity and contributing factors involved.

First, many participants noted a rise in sexual violence incidents, attributing this trend to the lack of punitive measures against offenders and the inadequacies within the judicial system. One community health worker from Mamou stated:

« … Punitive measures are very important … You must punish the perpetrators with heavy penalties to discourage those who do it … You also must recognize that the judiciary system is weak because perpetrators are often released by the court within some months… and the survivors of sexual violence can meet their aggressors in the neighbourhood … » **Community health workers, Mamou**

Second, participants identified various factors contributing to sexual violence against women and girls, including inappropriate dressing, multiple sexual partners, visiting leisure venues, substance abuse, frequenting high-risk areas (such as forests and isolated locations).

While some participants linked dressing style to increased vulnerability, other vehemently disagreed. A survivor of sexual violence from Mamou remarked:

« … Nowadays, when a girl dresses inappropriately, she exposes herself to violence as she draws boys’ attention towards her … All that the eye sees, the heart craves … » **Survivor of sexual violence, Mamou**

In contrast, a local NGO manager stated:

« … There is a tendency to relate the upsurge of sexual violence to the way girls dress… People forget that 7-month-old and 3-year-old babies are nowadays sexually abused … Dressing manner can never justify or be a source of sexual abuse! … **» Local NGO manager, Mamou**

Participants also acknowledged the psychological repercussions of sexual violence, highlighting that survivors often face stigmatization and rejection from their communities. One local NGO manager explained:

« … They [survivors of sexual violence] are always stigmatized … Even their families are stigmatized … They are often taken as bad examples… » **Local NGO manager, Mamou**

A health facility manager in Mamou also argued that survivors of sexual violence “lose their social value” and face difficulties in getting married.

Moreover, participants noted the severe health implications of sexual violence, including sexually transmitted infections, unwanted pregnancies, and even death.

#### Type of responses to sexual violence

Responses to sexual violence could be grouped into five district areas: individual, family, community, health system level responses, and responses from gender and women’ promotion structures.

#### Individual level

Most participants reported that survivors often choose to remain silent, especially when the perpetrator is a partner or family member. This silence is linked to fear of criticism from family or society. One security agent in Télimélé stated:

« … For many women, complaining to their husband is tantamount to divorce and creating an environment with her in-laws » **Security agent, Télimélé**

A local NGO manager in Mamou noted:

« … Sexual violence is prevalent in our society, but available data do not reflect this trend because women prefer keeping silent for fear of stigmatization… Society would ostracise women who take their husbands to court … » **Local NGO manager, Mamou**

#### Family level

According to participants, families typically respond to sexual violence by seeking conciliation or compromise, relocating the survivor, and pursuing compensation. The desire for conciliation is often rooted in fears of retaliation, social exclusion, and the potential for creating a hostile precedent with the perpetrator's family. A health provider commented:

« … If you take a sexual violence perpetrator to court, their family and society will view it as exposing your Children … In the future, if someone from your family commits a similar act, they will use this incident to revenge … » **Health provider, Mamou**

A religious leader in Mamou pointed out that families who report their daughter's sexual abuse are often blamed by the perpetrator's family for the arrest. In Télimélé, a health provider explained that survivors’ families often prefer “out-of-court settlements”, involving elders or religious leaders to mediate using socio-cultural or religious ties.

Concerns about breaking family ties also influence family responses. A security officer reported:

« … The aggressor is often a relative or a neighbour … [In such cases], the survivors parents disguise the situation, telling themselves that they have been together for decades and need to forgive and move on … They resist identifying or arresting the perpetrator … » **Security Agent, Mamou**

According to participants, families also fear social exclusion and stigma associated with sexual violence. A health provider in Télimélé noted:

« … In cases of sexual abuse, even the survivor's family often wants to keep the matter private. Widespread sharing of information can hinder the survivor chances of marriage in the communit … » **Health provider, Télimélé**

To mitigate stigma, families sometimes relocate to improve the survivor's prospects for marriage. Depending on the severity of the assault, families prioritize seeking care for the survivor:

« [When a sexual assault occurs,] the priority is first to provide care to the survivor. After that, we inform community structures against Gender-based violence … » **Health Center Manager, Mamou.**

Additionally, families often seek compensation, often with support from NGOs and gender and women’ promotion departments.

#### Community responses

Community responses to sexual violence often involve stigmatizing survivors, who are viewed as negative role models. Participants indicated that community members are proactive in supporting survivors immediately after an assault. This includes actively searching for the aggressor, especially if the assault occurred outside the community, such as in bushes or at night. One local NGO representative in Mamou stated:

 « We are ready to support, especially if it's someone close to them, by providing information to security and coordinating with OPROGEM to arrest the perpetrator. » **Local NGO, Mamou.**

Community members also assist survivors in accessing healthcare by providing transportation to medical facilities. A security agent in Mamou mentioned:

« … Sometimes, transport unions will send a driver to take the survivor and their relatives to the health facilities … » **Security agent, Mamou**

Another form of community support includes visits and financial aid during the survivor's hospital stay, which participants regarded as vital psychosocial support for both survivors and their families.

#### Health system responses

Interviews revealed that local health systems, including health centers and posts, offer emergency care to survivors before referring them to specialized facilities. Emergency care at peripheral health facilities focuses on treating physical trauma, while specialized facilities handle psychosocial support, sexually transmitted infection prevention, and pregnancy care. The Head of a Health Center in Mamou noted:

« This kind of violence [sexual violence] is frequent … Most often, the police refer survivors to us [in health facilities] … When we receive cases, our duty is to provide first aid, create a referral bulletin, and then refer the survivor to a district or regional hospital. **Head of Health Center, Mamou.**

#### Responses from gender and women’ promotion structures

Participants emphasized the significant role of gender and women’ promotion structures in identifying aggressors and assisting survivors in seeking compensation. The existence of deconcentrated structures for gender and women’ promotion, such as the Guinean child protection system, facilitates these efforts. These structures operate at various levels of territorial administration, including village child protection councils, local child and family councils, and district child protection coordination.

The district child protection coordination brings together stakeholders in basic social services (health, education, transport, justice, and security) and convenes monthly. Additionally, gender and women’ promotion structures support survivors in their pursuit of compensation. An NGO representative in Mamou stated:

« We are ready to assist the survivor … If the aggressor is someone close, we provide information to the police so that the family is not ostracised by the community. » **representative of local ONG**, **Mamou**

#### Challenges experienced by stakeholders in addressing sexual violence

Stakeholders involved in *n* combating sexual violence encounter various challenges at the family, community, and health system levels.

#### Family-Level challenges

Participants reported socio-cultural and economic barriers that hinder survivors’ access to health services. Many families hold the belief that seeking help will lead to the exposure of their daughters’ identities, driven by fears that authorities, such as the police or health services, will disclose sensitive information to the community. As noted by a representative from the Directorate of gender and women’ promotion in Télimélé.

« … Parents sometimes think that by using health services, the authorities will be informed, and their daughter will be exposed … » **directorate of gender and women’ promotion, Télimélé**

Additionally, families face economic burdens when seeking healthcare for survivors. These barriers encompass both direct costs (e.g., medical fees, medication) and indirect costs (e.g., transportation for referrals, food, and other expenses associated with hospital stays).

#### Community-level challenges

At the community level, participants highlighted the ineffective functioning of community-based GBV structures, especially in rural and remote areas. This inadequacy limits access to information regarding the existence and services of these structures. Key challenges include a lack of logistics, financial resources, and trained personnel. A representative from the Directorate of gender and women’ promotion in Télimélé stated:

« … At sub-district level, there's the local child protection committee, at district level there's the local child and family council, and at village level, there's the village child protection council … In most districts and villages, these structures rely on volunteers … If they are not motivated, they won't feel obliged to work … » **directorate of gender and women’ promotion, Télimélé**

#### Health system-level challenges

At the health system level, challenges faced by stakeholders include the lack of integrated care for sexual violence, the frequent stock-outs of medical products, and the shortages of specialised and skilled health professionals for the management of sexual violence.

Participants indicated that the care package for sexual violence does not adequately cover related costs, such as transportation and food, impacting the quality of care provided by health professionals. Although national health policies recommend free care for sexual violence survivors, procurement delays for treatment kits mean that only half of hospitals’ needs are met. This often results in financial constraints for specialized health facilities, leading to the implementation of free care policies for conditions like malaria and cesarean sections, further straining resources.

Consequently, many survivors and their families face direct payments for care, which contributes to delays in seeking treatment and underutilization of healthcare services for sexual violence. The lack of competent health personnel in peripheral facilities means that psycho-social support services are primarily available only at specialized health centers.

## Discussion

This situational analysis provided a comprehensive understanding of the frequency, perceptions, responses and key challenges faced by stakeholders in addressing sexual violence in rural Guinea. Sexual violence accounted for more than half of all reported cases of GBV. Survivors and their families often sought conciliation with the perpetrators’ families due to fear of retaliation, stigmatization and social exclusion. The findings also highlight the lack of integrated care for survivors of sexual violence within the Guinean health system.

This study revealed a high proportion (55%) of sexual violence out of all GBV in these two rural districts of Guinea. According to global estimates, SSA accounted for 37% of sexual violence cases among girls and women in 2018 (5). In comparison, Guinea shows a disproportionately high frequency of sexual violence. In 2017, sexual violence comprised 23% of all GBV cases, which is 2.4 times lower than the prevalence observed in this study in 2020 (37). A plausible explanation for this increase in reported sexual violence may relate to the reporting system employed. Our study utilized routine data, which did not capture most of the female genital mutilation (FGM) cases. Given that FGM constitutes 90%–95% of all GBV cases, its underreporting—largely due to cultural normalization—may have led to an inflated proportion of other GBV forms, including sexual violence (33, 37). The absence of a universal instrument for detecting and reporting GBV globally is known to undermine their prevalence (40, 41). Without standardized tools, data collection methods can vary widely, leading to inconsistencies in prevalence rates (41, 42). As highlighted in the literature, discrepancies in definitions, methodologies, and cultural contexts can further complicate efforts to accurately assess and address GBV worldwide (40–42). Developing a universally accepted instrument could enhance the reliability of data on GBV, facilitate cross-country comparisons, and ultimately inform more effective interventions (43). Another potential factor contributing to this rise could be improvements in the reporting system due to incremental efforts in raising community awareness and enhancing the capacities of community structures involved in GBV prevention and response (44, 45).

However, the overall proportion of sexual violence conceals significant discrepancies in data reporting between districts. For example, in Mamou, sexual violence accounted for 61% of GBV cases, compared to only 8% in Télimélé. These findings suggest that substantial efforts are still required to address GBV and sexual violence in Guinea, starting with the standardization of data reporting tools. Disparities in data collection methods and terminology further complicate the interpretation of GBV cases. For example, categories used by health facilities such as “child GBV” and “female GBV” fail to specify whether the violence was sexual or non-sexual violence.

Improving the GBV reporting process also requires enhanced communication and coordination between key stakeholders involved in combatting sexual violence in Guinea. This could be achieved by revising GBV reporting standards and procedures and providing targeted training healthcare providers. A clear and consistent typology of sexual violence, aligned with international classifications —such as “attempted rape”, “rape”, and “sexual harassment”— should also be adopted (6, 12). Strengthening the coordination of GBV interventions at the national level is critical. This includes appointing dedicated resource personnel within the Ministry of gender and women’ promotion, tasked with centralizing data, overseeing case management, and ensuring the provision of quality care and follow-up for survivors.

Moreover, conciliation emerged as the predominant response adopted by survivors of sexual violence and their families. This practice, corroborated by other studies including, including in Ethiopia, is driven by fears of retaliation from perpetrators or their families, particularly in contexts where law enforcement is weak, and perpetrator may be released after only a few months of imprisonment (31, 46, 47). Beyond the risk of retaliation, this practice exerts continuous psychological pressure on survivors, increasing their vulnerability to further sexual violence and negatively impacting their mental health (48–50). Another key factor driving survivors and their families toward conciliation is the fear of social stigmatization and rejection. These findings reflect the complex social dynamics surrounding sexual violence. Based on practical experience, the inadequacies in legal proceedings for sexual violence —such as the premature release of perpetrators—are partially attributable to the low evidentiary value of medical certificates issued at district levels, including in our study areas. This issue is compounded by the absence of specialised personnel in these districts, leading to delays in the issuance of medical certificates, which are often produced by health professionals untrained in forensic documentation. Such unofficial certificates carry limited legal weight and are easily contested by perpetrators’ legal representatives, making it difficult to establish a direct link between the violence and the accused. Additionally, the limited enforcement of laws pertaining to sexual violence exacerbates the challenges survivors face in seeking justice. It is thus crucial to strengthen the implementation of legal frameworks related to GBV in Guinea, including sexual violence.

This study further underscores the lack of integrated care for survivors of sexual violence in Guinea. Survivors and their families face significant financial burdens, both direct and indirect, in accessing healthcare services. Direct costs include expenses for medications and care, often exacerbated by frequent stock-outs of essential medical supplies. Before reaching referral facilities, survivors are also required to make out-of-pocket payments for emergency services at primary healthcare centers. Additionally, they bear indirect costs such as transportation, food, and expenses associated with prolonged hospital stays in referral health facilities. These findings highlight the urgent need to improve healthcare access for survivors of sexual violence, advocating for the integration of GBV services within health facilities. This is particularly pertinent given that a significant portion of Guinea's rural population (58%) lives below the poverty line. Preliminary findings from this situational analysis contributed to the adoption of a results-based financing strategy that includes free healthcare for sexual violence survivors. Ongoing research on the implementation and outcomes of the results-based financing initiative will provide further insights into how this approach enhances referral pathways and improves access to quality healthcare for survivors of sexual violence (48).

## Study strengths and limitations

To the best of our knowledge, this is one of the first mixed-methods study studies in Francophone West Africa to examine the frequency, perceptions, responses, and challenges experienced by stakeholders in addressing sexual violence. The study's implementation and reporting adhered to the internationally recognized Consolidated Criteria for Reporting Qualitative Research Guidelines (51). However, several methodological limitations should be acknowledged. This study was conducted in only two out of 38 districts in Guinea, which may limit the generalizability of findings related to the number of reported cases of sexual violence at the national level. Nonetheless, the mixed-methods design, particularly the qualitative approach, enhances the transferability of findings concerning stakeholders’ perceptions, responses, and challenges (52).

## Conclusion

The study data revealed a high frequency of sexual violence in Guinea, with notable variations between the two districts and among different data sources. This situational analysis provided valuable insights into the perceptions, responses and challenges faced by stakeholders in addressing sexual violence in in the country. Responses from survivors and their relatives predominantly involved seeking conciliation with perpetrators’ families, driven by fear of retaliation, stigmatization, or social exclusion. However, significant barriers to accessing healthcare services for survivors, such as socio-economic and cultural constraints, insufficient human, material, and financial resources, further exacerbate the challenges in managing sexual violence in Guinea.

This finding underscores the need for stakeholders to focus on reinforcing the capacities of decentralized services involved in combatting sexual violence. This includes improving the motivation of service providers, particularly by offering stable employment to volunteer healthcare workers in peripheral health structures. There is also a critical need to strengthen the enforcement of laws on sexual violence and improve the affordability and accessibility of quality, integrated healthcare services for survivors. Ensuring confidentiality and providing comprehensive care—such as improving the availability of medical products, and access to psycho-social services—are essential steps forward. Moreover, harmonizing data reporting tools for GBV, including sexual violence, would help streamline efforts across Guinea. Finally, addressing the shortage of skilled healthcare professionals responsible for issuing medico-legal certificates at the district level is crucial for improving legal outcomes and care for survivors of sexual violence.

## Data Availability

The original contributions presented in the study are included in the article/Supplementary Material, further inquiries can be directed to the corresponding author.
